# Large vestibular schwannomas presenting in the late state of pregnancy: a case report and literature review

**DOI:** 10.3389/fneur.2023.1270989

**Published:** 2023-12-01

**Authors:** Mingbin Bao, Yunsen He, Ye Tao, Li Liu, Yuheng Li, Yongjun Zhu, Qinjiang Huang, Mengjun Zhang, Bo Wu, Hao Wang

**Affiliations:** ^1^Department of Neurosurgery, Sichuan Provincial People’s Hospital, University of Electronic Science and Technology of China, Chengdu, China; ^2^Department of Neurosurgery, Sichuan Lansheng Brain Hospital & Shanghai Lansheng Brain Hospital Investment Co., Ltd, Chengdu, China; ^3^Department of Imaging, Sichuan Provincial People’s Hospital, University of Electronic Science and Technology of China, Chengdu, China; ^4^Department of Neurosurgery, Suining Municipal Hospital of TCM, Suining, China; ^5^Department of Neurosurgery, Wenjiang District People’s Hospital of Chengdu, Chengdu, China; ^6^Department of Psychiatry, Sichuan Provincial Center for Mental Health, University of Electronic Science and Technology of China, Chengdu, China; ^7^Department of Neurosurgery, Jianyang Chinese Medicine Hospital, Chengdu, China

**Keywords:** neuroma, pregnancy, vestibular schwannoma, surgery, multidisciplinary

## Abstract

Vestibular schwannomas in pregnancy have rarely been reported, and there is a lack of in-depth discussion on the experience of management of massive acoustic neuromas in pregnancy. Herein, we present a pregnant woman with a giant vestibular schwannoma and obstructive hydrocephalus who presented at 30 weeks of gestation. She was initially misdiagnosed as having a pregnancy-related reaction of headache, dizziness, and vomiting that had occurred 2 months earlier. After observation at home, her symptoms progressed at 30 weeks of gestation, and imaging findings revealed a brain tumor in the CPA region with secondary cerebella tonsil herniation and obstructive hydrocephalus, and she was transferred to our center for treatment. Consequently, we relieved her hydrocephalus with a ventriculoperitoneal shunt (V-P shunt) and used corticosteroids to simulate fetal maturation. After 10 days, her mental condition deteriorated, and her right limb muscle strength gradually decreased until grade 0 (MMT Grading). Finally, under a joint consultation with the Department of Neurosurgery, Obstetrics, and Anesthesiology, she underwent a cesarean section under general anesthesia and first-stage tumor removal at 31 weeks of gestation. Upon discharge, the previously observed neurological deficits, which were reversible and had manifested during her gestational period, had been successfully resolved, and the fetus had been conserved. The neuroimaging confirmed the complete tumor removal, while the neuropathologic examination revealed a vestibular schwannoma. Therefore, we recommend early diagnosis and treatment for these patients, especially people with headaches, vomiting, and sudden hearing loss during pregnancy. Herein, we concluded that our cases provide a valuable experience in the latest acceptable time frame for the operation to prevent irreversible neurological impairment and premature delivery in late pregnancy.

## 1 Background

Vestibular schwannoma (VS) is a common slow-growing benign lesion located in the cerebellopontine angle (CPA) region and originates from the Schwann cell. Pregnant women with VS are at a significantly increased risk of abortion and death compared to their non-pregnant counterparts (1). During pregnancy, the incidence of vestibular schwannomas is infrequent, with a global report of only 37 cases (Table 1). Although VS normally grows slowly, during pregnancy, due to increased estrogen levels and enhanced hemodynamics are thought to stimulate rapid tumor growth (≥4 mm/year) that results in rapidly progressive symptoms and even lead to acute obstructive hydrocephalus (2–6). Empirical evidence indicates that the mean size of vestibular schwannomas (VS) in pregnant individuals exceeds that of non-pregnant counterparts (5, 7). Additionally, in previous cases of pregnancy with VS, the tumor also tended to be large (>4 cm; Table 1). As a result of the “irrigation effect” of estrogen on VS during pregnancy, the tumor often progresses rapidly and is frequently diagnosed at a large size (3, 8, 9). Certain patients who are diagnosed with a vestibular schwannoma during the initial stages of pregnancy choose conservative observation. However, it has been observed that the neuroma may exhibit rapid growth during pregnancy, leading to the exacerbation of symptoms in the later stages of pregnancy (9, 10). Additionally, VS patients in late pregnancy are at great risk for the smooth administration of anesthesia, fetal protection, cesarean section, and tumor removal. This presents a serious challenge for the fields of neurosurgery, obstetrics, and anesthesiology.

**Table 1:** List of previous cases of pregnant women with vestibular schwannomas.

Authors and year	Age, G/P	Diagnosis (weeks)	Tumor size (cm)	Symptoms	Clinical progression (weeks)	Delivery (weeks)	Tumor resection (stage)
Doyle et al. (2)	28, G1/P0	8–9	2	HD	None	Term	T2
Doyle et al. (2)	29, G1/P0	11	4.5	HD	None	Term	T2
Kachhara et al. (8)	27, G2/P1	22	4.5 × 4	HA, VO, HD, GD, FD, DI	10	Term	T2
Satyarthee (23)	37, ukn	18	4 × 3.9 × 3.6	HD	None	Term	T2
Thacker et al. (10)	27, G1/P0	31	Large	VO, GD, VD, HD	31	38	T3
Beatty et al. (12)	33, ukn	28	4	ukn	ukn	Term	T3
Akella et al. (9)	21, G1/P0	BD	4.0 × 2.4	HD, VD	25	34	T3
Gaughan et al. (7)	30, G2/P1	20	4 × 4	VO	ukn	Term	DP
Kachhara et al. (8)	30, G2/P1	36	4	HA, VO, GD	32	36	AD
Magliulo et al. (26)	24, G1/P0	24	4.5 × 4	HD, HA, VO	24	Term	AD
Shah et al. (3)	20, G1/P0	26	4.7 × 3.4	GD, HD, VO, VD, LW	27	27	AD
Bedard et al. (24)	30, G2/P1	30	3.8 × 3.8 × 3.8	FD, HD	none	36	AD
Beni-adani et al. (21)	24, G4	35	6	HA, HD, AT, HE, DA, DP, DY	35	37	AD
Kurowska-Mroczek., (4)	33, ukn	30	6	VD	Ingravescence	33	AD
Hsiao et al. (31)	30, G2/P1	30	4.5 × 4	HD, HA, VO, DI, GD, DA	36	36	AD
Allen et al. (11)	16, G1/P0	AD		HD, FD	24–25	Term	AD
Allen et al. (11)	21, G1/P0	AD		HD	None	Term	AD
Allen et al. (11)	ukn, G2/P0	AD		HD, HA, VO	None	Term	AD
Allen et al. (11)	24, G3	AD		HD	T3	Term	AD
Allen et al. (11)	31, G4	AD		HD	None	Term	AD
Allen et al. (11)	36, G2	AD		HA, FD, TI,	20–21	Term	AD
Allen et al. (11)	24, ukn	AD		HD	None	Term	AD
Allen et al. (11)	34, ukn	34		AT, FD, DA, HD	T3	36–40	AD
Sharma et al. (32)	23, G1/P0	38	3.9 × 3.4	HA, HD, FD	38	38	AD
Moafi et al. (20)	27, ukn	36	5.1 × 2.3	VO, HD, FD	none	40	AD
Present case	25, G1/P0	30	6 × 5	HA, DIZ, VO LW, GD,	30	31	AD
Beatty et al. (12)	34	ukn	2.5	ukn	ukn	ukn	AD
Beatty et al. (12)	29	ukn	4.5	ukn	ukn	ukn	AD
Beatty et al. (12)	33	ukn	3.5	ukn	ukn	ukn	AD
Beatty et al. (12)	28	ukn	3	ukn	ukn	ukn	AD
Beatty et al. (12)	23	ukn	3	ukn	ukn	ukn	AD
Gaughan et al. (7)	ukn	ukn	5.5	HA, TI, VO, VD, GD	ukn	ukn	AD
Gaughan et al. (7)	ukn	ukn	4.5	HA, TI, HD, FD, VO, GD, DI	ukn	ukn	AD
Gaughan et al. (7)	ukn	ukn	4	HA, TI, HD, FD, VO, GD	ukn	ukn	AD
Gaughan et al. (7)	ukn	ukn	5	HD, HA, DI	ukn	ukn	AD
Gaughan et al. (7)	ukn	ukn	3	HD, GD	ukn	ukn	AD

ukn, unknow; AD, after delivery; HA, headache; HD, hearing disturbance; VO, vomiting; GD, gait disturbance; FD, facial disturbance; DI, diplopia; VD, visual disturbance; LW, lib weak; AT, ataxia; HE, hemiparesis; TI, tinnitus; DIZ, dizziness; DY, dysphagia.

In this report, we presented a case of a woman in the late stage of pregnancy who also exhibited symptoms of VS. This case presented the largest VS identified in pregnant women until now. Moreover, it represents the only case in which the patient presented with obstructive hydrocephalus and progressive neurological deficits while successfully attempting to preserve fetal and all neurological functions. The objective of this study is to examine the optimal timing of surgical intervention for women diagnosed with vestibular schwannoma and obstructive hydrocephalus during late pregnancy. Additionally, this study aims to explore the potential benefits of a multidisciplinary approach to treatment and surgical intervention for vestibular schwannoma in pregnant women.

## 2 Presentation

### 2.1 Basic information of the pregnant

**Figure 1:**
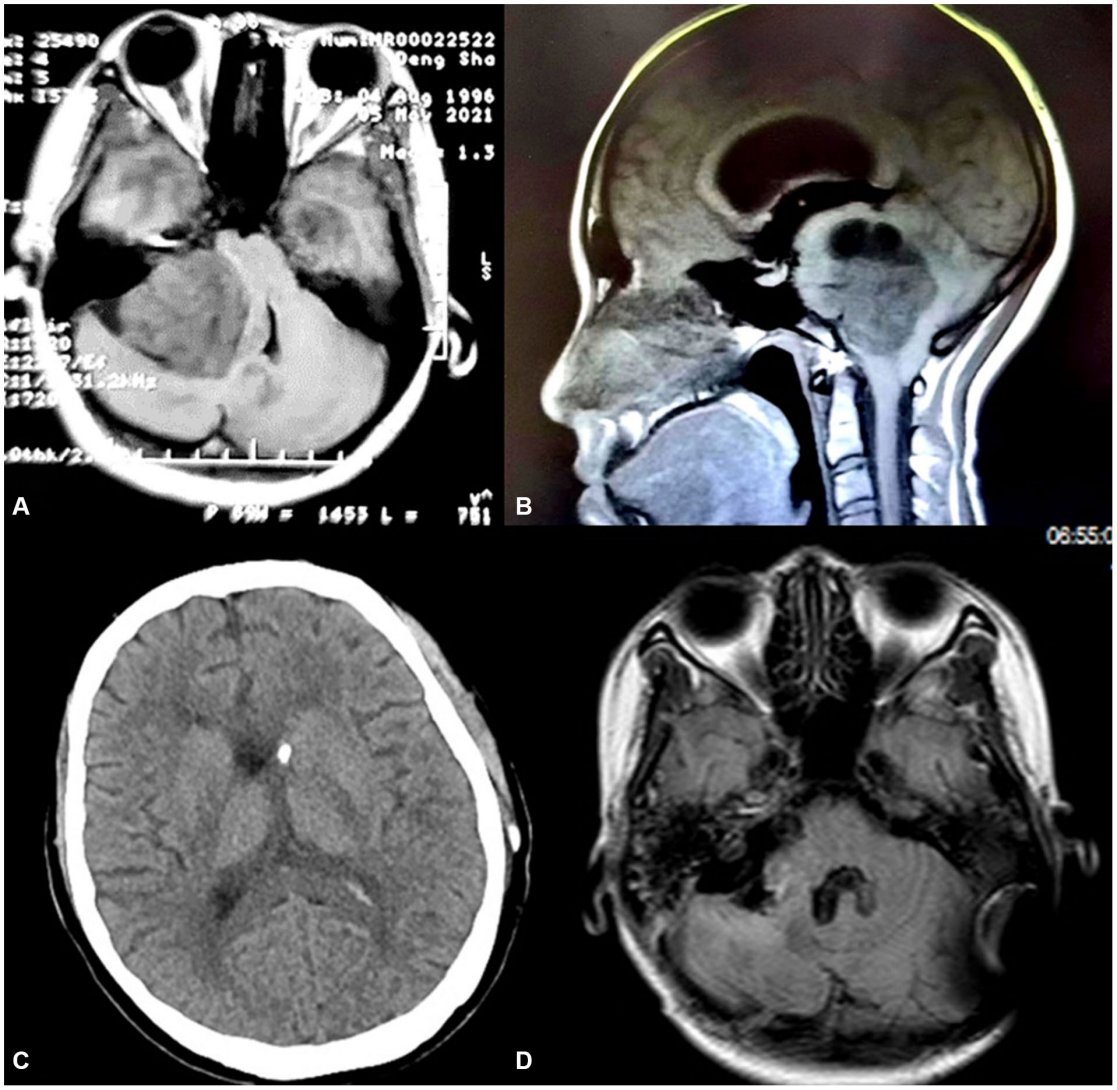
**(A,B)** There is a large solid mass about 6*5 cm in size in the right CPA area with ventricular and brainstem compression; **(C)** After the V-P shunt, CT revealed significant relief of her hydrocephalus; **(D)** Postoperative magnetic resonance imaging shows complete removal of the tumor.

A 25-year-old woman at 30 weeks gestation was transferred to our center with paroxysmal headaches, dizziness, and vomiting that persisted for 2 months. Initially, she consulted the obstetrics department at a local hospital, where she was misdiagnosed as having a pregnancy reaction, and subsequently decided to monitor her condition from home. Two months later, she went to the neurosurgery department in a local hospital due to deteriorating symptoms, including recent blurred vision and limb weakness. Subsequently, a tumor in the cerebellopontine angle region was detected by cranial MRI (Figure 1).

### 2.2 Physical examination of the nervous system

Upon admission, the patient underwent a neurological examination which revealed that she was conscious, with approximately grade 3 (MMT Grading) muscle strength in the right leg. Additionally, the patient presented with hearing loss on the right side. There were no other neurological signs, including diplopia, ataxia, or posterior cranial nerve disorders. Pure-tone audiometry showed a significant loss of sensorineural hearing on her right side. Moreover, the abdominal examination shows that the maternal uterus is of a size consistent with pregnancy, and no uterine contractions are palpable. The fetal presentation was cephalic, and the fetal heart rate was recorded at 128 bpm.

### 2.3 Imaging for brain

Magnetic resonance imaging (MRI) revealed a large solid tumor in the right CPA region with a size of 6*5 cm, which caused obstructive hydrocephalus and herniation of the cerebellar tonsils (Figures 1A,B).

### 2.4 Examination of the fetus

At 30 weeks gestation, the fetal ultrasound showed a pregnancy-sized fetus with a biparietal diameter of 7.8 cm and a femoral diameter of 5.4 cm. The fetal position was on the head position, and the fetal heart rate was 154 bpm. There was no umbilical cord around the neck of the fetus, and the placenta was mainly located in the anterior wall. The placenta function was grade 1 (Grannum grading), and the umbilical artery blood flow was normal.

### 2.5 Treatment strategy

The patient was transferred to our center for treatment, after obstetric consultation, a fetal ultrasound indicated that fetal growth was 1 week below gestational age. Consequently, corticosteroid treatment was administered to promote fetal lung maturation in anticipation of perioperative delivery.

She refused to undergo immediate surgery and instead preferred to postpone tumor excision until postpartum. Subsequently, we consulted anesthesiologists, obstetricians, and neonatologists about our treatment plan and proposed an extension of the gestational period to 34–36 weeks subject to favorable circumstances.

Following the placement of a ventriculoperitoneal shunt during the 30th week of gestation, she was subsequently transferred to the intensive care unit (ICU) for ongoing management. An immediate postoperative computed tomography scan (CT) revealed the presence of hydrocephalus, apparent ventricular dilatation, and brainstem compression. Moreover, she described significant decrease in her headache and blurred vision.

**Figure 2:**
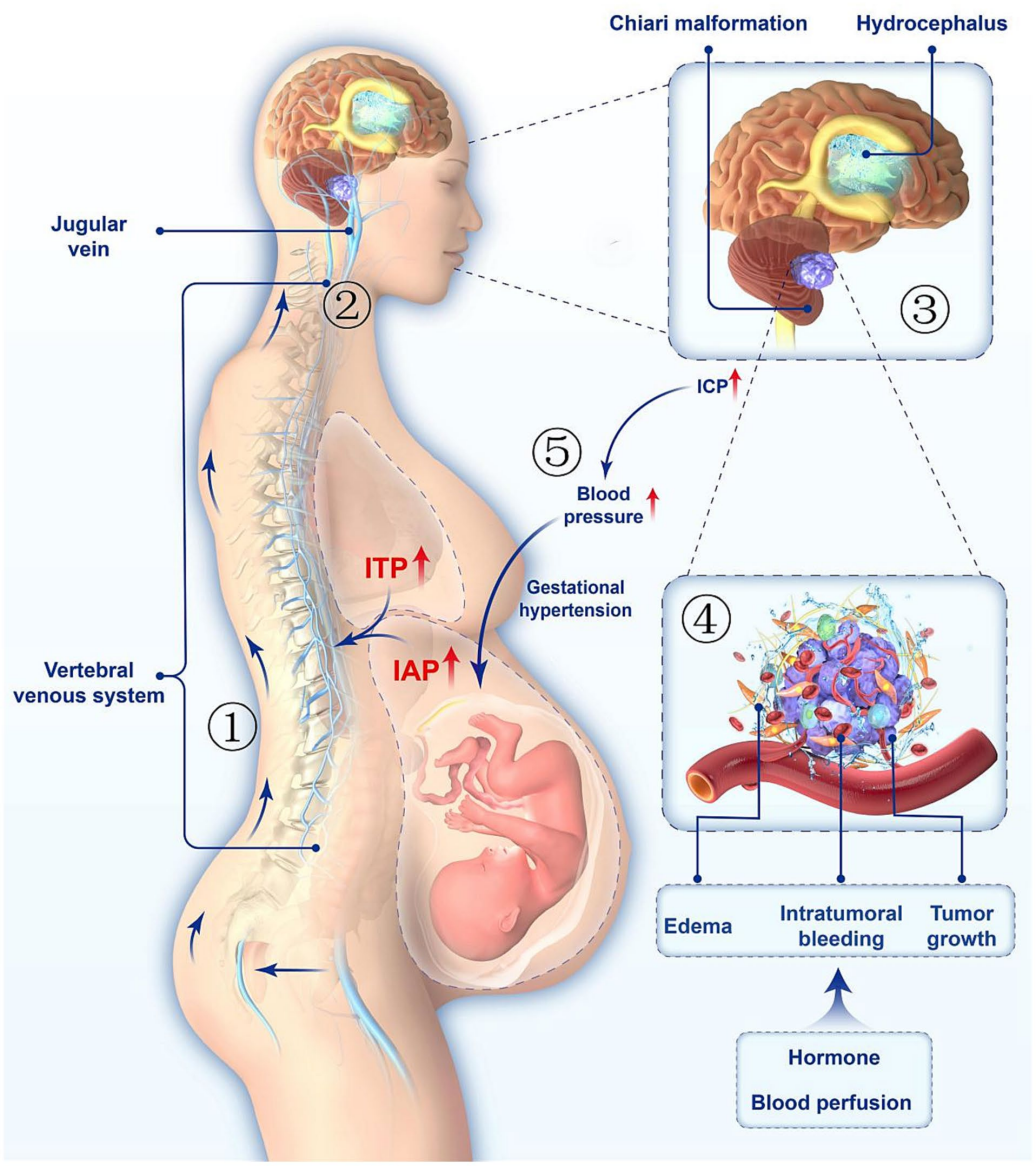
Pathophysiological changes in the body of a pregnant woman with an vestibular schwannoma. ① Increased intra-abdominal pressure raises intracranial pressure by causing flow of venous blood into the brain through intra-vertebral venous congestion. ② Increased intra-abdominal pressure and secondary increase in intrathoracic pressure, resulting in increased intracranial pressure due to obstruction of jugular venous blood backflow. ③ Tumor in the intracranial CPA region causing compression of the cerebrospinal fluid outflow tract, resulting in secondary hydrocephalus and subcerebellar tonsillar herniation. ④ Increased perfusion and sex hormones may be important factors in causing tumor oedema, intratumoural bleeding and accelerated tumor growth. ⑤ When ICP is elevated, the automatic adjustment of blood pressure further aggravates the gestational hypertension.

After 3 days, the state of consciousness turned to a state of drowsiness, and there was a progressive decline in the muscular strength of the right limb. Ten days later, her right limb strength was observed to have decreased to level 0. Furthermore, the follow-up CT scan showed a significant improvement in her hydrocephalus (Figure 1C). However, it was observed that the brainstem was severely compressed (Figure 2). After another comprehensive assessment by the aforementioned departments, she underwent urgent cesarean delivery at 31 weeks gestation while under general anesthesia. The neonate was born alive and received an Apgar score of 8. Subsequently, the neonate was transferred to the neonatal ICU for additional medical attention.

Following the cesarean section, the tumor was removed through a suboccipital retrosigmoid approach. The operation was successful as it showed a 6 cm diameter solid tumor with good blood supply, during which her facial nerve was well preserved.

Immunohistochemistry examination of the tumor showed a positive result for CD34, indicating an significant blood supply and diffuse positivity for S-100 and SOX10, thus confirming the diagnosis of vestibular schwannoma.

### 2.6 Postoperative

On the first day following the operation, the patient exhibited a muscle strength of grade 3 in the right limb and regained consciousness. By the fourth day, the patient’s muscle strength in the right limb had improved to grade 4. During the three-month follow-up post-discharge, we observed that the baby was in good condition, the patient had fully regained muscle strength in her right limb, and her facial nerve function was at level HB1, with the exception of experiencing difficulty in recovering hearing on the right side. Furthermore, MRI scan indicated the absence of hydrocephalus and cerebella tonsil herniation, and the tumor was completely removed (Figure 1D).

## 3 Discussion

### 3.1 Mechanistic and hormonal mechanisms for accelerated growth of vestibular schwannoma

Higher levels of hormones during pregnancy, especially in the late trimester, may be a factor in promoting tumor growth (11). However, the role of estrogen in VS has not been clearly identified (12–15). Due to the increased blood volume, increased jugular vein pressure and estrogen levels during pregnancy, the tumor is at a higher risk of exacerbation related to increased edema, peritumoral effusion, and the formation of intratumoural bleeding (11, 16). Previously, it has been observed that acoustic neuromas presenting during pregnancy tended to be larger, suggesting that the biology of the tumor may be altered during pregnancy, resulting in an increased risk of tumor progression. Therefore, further studies are needed to explain the mechanism of VS progression.

### 3.2 Due to its symptoms resembling pregnancy reactions, it is prone to misdiagnosis

**Figure 3:**
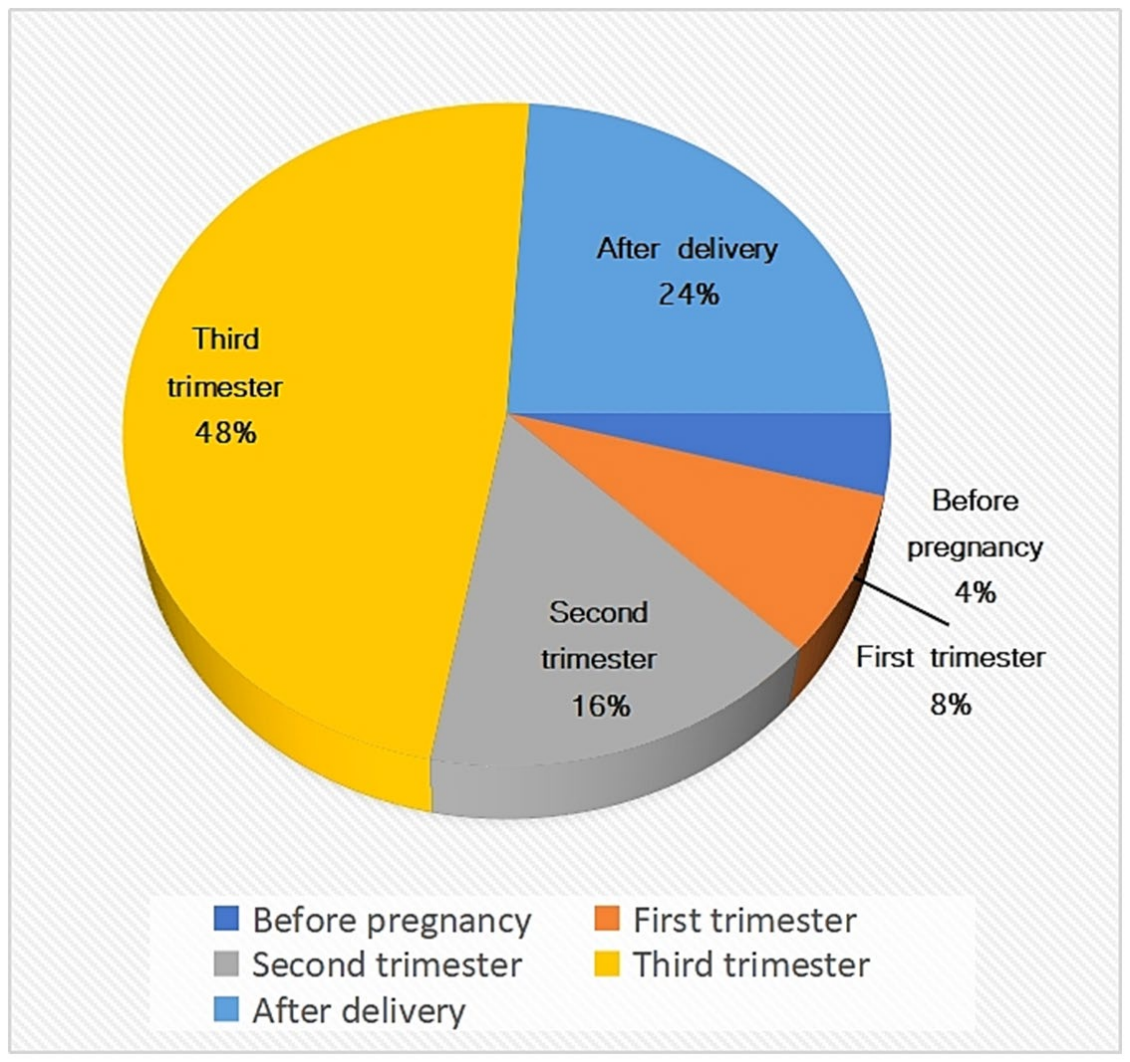
Statistical chart about the period when vestibular neuroma is detected in pregnant women.

Previously, half of the patients were already in late pregnancy at the time of presentation, and only 8% were found to have VS in early pregnancy (Figure 3). Therefore, the high rate of underdiagnosis of this disease deserves our attention. Nausea and vomiting are not specific to pregnancy, and new occurrences of nausea and vomiting in the middle and late stages of pregnancy should be carefully examined to exclude VS (17). Pregnant women can also experience sudden hearing loss during pregnancy. According to previous clinical presentations, it has been observed that over 80% of expectant individuals who experience sudden sensorineural hearing loss (SSNHL) typically manifest the syndrome during the mid or late stages of pregnancy. This occurrence may be attributed to an increase in sex steroid hormone levels, embolism of the labyrinthine artery due to maternal hypercoagulability, obstruction of the cochlear microcirculation, or autoimmune diseases (18, 19). SSNHL occurred during pregnancy and presented with moderate to severe sensorineural hearing loss. The clinical presentation of the condition is characterized by asymmetrical hearing loss, accompanied by occasional tinnitus and vertigo, which typically remit upon the end of gestation. Approximately 15% of SSNHL patients exhibit symptoms of vestibular schwannoma. Moreover, It has been observed that some obstetricians may neglect the occurrence of sudden hearing loss in pregnant patients with this condition (19). Consequently, a comprehensive history collection and thorough physical examination are imperative. In cases of sudden hearing loss, it is recommended to conduct neuroimaging of the brain and inner ear as a means of excluding VS (19, 20).

### 3.3 The surgery time for VS with a pregnant

The co-occurrence of VS and pregnancy is an infrequent phenomenon, thus necessitating a careful assessment of the optimal timing for surgical intervention that considers the potential harm to both the mother and the developing fetus.

### 3.4 Vestibular schwannomas in the first trimester (1–12 weeks)

Non-obstetric surgery is not recommended in early pregnancy because anesthetic drugs and stress caused by surgery tend to affect the fetus. Patients at this stage are often asymptomatic or have mild symptoms that they themselves attribute to pregnancy. Consequently, reports are infrequent, with only about 8% of cases being identified as VS in early pregnancy (Figure 3). While considering treatment measures for VS in early pregnancy, the mother’s wishes should be given priority. It has been suggested that patients diagnosed with VS early in pregnancy should be conservatively observed, with V-P shunts or emergency surgery should only be considered in cases of hydrocephalus or more severe neurological deterioration (3), given their potential fetal implications. Therefore, it is crucial for the mother to be actively involved in the decision-making process, as she must face the potential reality of terminating the pregnancy.

### 3.5 Vestibular schwannomas in the second trimester (13–24 weeks)

The best time to remove the tumor before delivery is in the middle of pregnancy when the possibility of fetal malformation or miscarriage due to anesthetic drugs is minimal, and the hormonal levels and hemodynamics of the mother have not significantly changed (3, 9, 21, 22). It has been suggested that removal of the tumor in mid-pregnancy may be considered for patients presenting with obstructive hydrocephalus and progressive neurological deterioration (23). In contrast, observation and follow-up are recommended for patients with mild symptoms and small tumors (3, 23). Doyle operated on two VS patients (4.5 and 2.0 cm in diameter) in mid-pregnancy without hydrocephalus who had only varying degrees of hearing loss and whose neurological function was preserved after the operation and eventually delivered successfully (2). Overall, four previous VS women underwent surgery in mid-pregnancy, three of whom were neurologically stable and did not have hydrocephalus (2, 8, 23) (Table 1). According to reports with complete clinical records, some patients underwent successful conservative observation for stable symptoms (11, 20, 24). However, there was a deterioration of symptoms in 59.1% of patients in late pregnancy (Table 1). (Among the 22 patients who underwent surgery during late pregnancy or postpartum, 13 experienced exacerbation of pre-existing neurologic disorders and new symptoms during late gestation.) According to the EANO guidelines for managing VS, surgery is the only treatment option for VS with brain compression (Koos IV) or larger than 3 cm in diameter (recommendation level is a good practice point) (25). To avoid rapid progression of VS during pregnancy, which increases the difficulty and risk of treatment of the patient, it is recommended that surgical intervention be conducted during mid-pregnancy for cases of solid tumors with a Koos classification of grade IV or a diameter exceeding 3 cm, irrespective of the presence of obstructive hydrocephalus in the pregnant patient. This is particularly pertinent for cases exhibiting cystic degeneration.

### 3.6 Vestibular schwannomas in the third trimester (25–36 weeks)

Typically, approximately 50% of the patients are in the advanced stages of pregnancy upon the diagnosis of VS. It is an unsuitable time for non-obstetric surgery due to the hemodynamic changes, high risk of intraoperative bleeding, and postoperative complications for the mother and fetus. Therefore, we recommend that pregnant women with VS receive medical attention in large general hospitals that offer a multidisciplinary treatment approach, with a primary focus on neurosurgery, to ensure the safety of the pregnancy process.

Delivery before resection is equally risky, as uncontrolled bleeding during delivery will impair the automatic intracranial adjustment and then cause a drop in cerebral perfusion pressure of the mother. In case of a successful delivery, conditions are improved for oncological surgical procedures.

To avoid the surgical risk brought by tumor edema and congestion, resection is reported to be performed 3–14 days or longer after delivery. In our case, when the patient presented with a significant decrease in neurological function and her muscle strength on the affected side was reduced to grade 0, we immediately resorted to a simultaneous cesarean section and tumor resection in the CPA region, after which the neurological function of the patient was rapidly restored. There is no objective method of predicting whether damage from tumor compression can be recovered. If surgical intervention is done before the decline of muscle strength reaches zero, it can still result in substantial recovery of the patient’s neurological function, as was in our case Therefore, if a rapid decline in muscle strength on the affected side is observed, or severe posterior group cranial nerve deficits develop, we recommend immediate operative intervention, especially if the conservative management strategy is adopted.”

It is recommended to maintain pregnancy until term for patients exhibiting stable symptoms during the latter stages of pregnancy, while ensuring that maternal health indicators remain stable and neurological function remains uncompromised. The patients who developed neurological symptoms in late pregnancy have been reported in the literature to have undergone VS resection after delivery, with good postoperative maternal and fetal outcomes (3, 7, 11, 26). This proves that conservative treatment measures and appropriate postponement of surgery, even until full-term delivery, are feasible and more conducive to newborn survival in cases with mild neurological dysfunction (27).

It is imperative to pay special attention to symptoms caused by tumor compression, including hearing loss, tinnitus, facial nerve, trigeminal nerve dysfunction, balance instability, ataxia, nystagmus, and headache, as these are prevalent in almost 50% of people. The manifestation of secondary hydrocephalus and sub-microcephalic tonsillar herniation resulting from the mass effect of the tumor includes a range of symptoms such as headache, vomiting, suboccipital pain and vertigo, and hearing loss. These symptoms may develop due to compression of the posterior group cranial nerve or brainstem (28). Headache associated with increased intracranial pressure is usually gradual; it worsens as time passes and is accompanied by a Valsalva maneuver that increases cranial pressure (29). The increased intra-abdominal pressure in the later stages of pregnancy may increase ICP through the congestion of intravertebral veins and obstruction of cerebral venous blood return (30). The elevated ICP and the increased blood volume in late pregnancy can further cause hypertension in pregnancy (Figure 2). Effective surgical decompression can significantly relieve symptoms caused by hydrocephalus and secondary hypertension to appropriately delay the delivery (3, 21). Based on the analysis of Kushal’s and other relevant cases, it is suggested that patients experiencing obstructive hydrocephalus in late pregnancy should undergo V-P shunts. However, in cases where the V-P shunt proves to be ineffective, immediate surgical decompression is recommended.

## 4 Highlights

1. In this case, the tumor exhibited the greatest diameter during advanced pregnancy. Furthermore, the patient had a good functional restoration after the surgical intervention.
2. Thanks to a multidisciplinary approach, we successfully completed a cesarean section and a tumor resection in the CPA region. Simultaneously, we prevented further neurological deficits, thereby protecting the patient’s neurological functionality. Therefore, it is recommended that these patients receive treatment at large general hospitals.
3. Based on a comprehensive analysis of previous cases, we are presenting recommendations regarding the optimal treatment modalities and the most appropriate surgical timing for patients with VS during different trimesters of pregnancy.

## 5 Conclusion

In this report, we have presented a case of a patient who experienced a late pregnancy combined with a large VS, resulting in significant neurological impairments. However, the patient was able to deliver the fetus successfully and achieve full neurological recovery with effective intervention. It is recommended that obstetricians exercise caution in the early diagnosis of pregnant women who present with symptoms of headache, vomiting, and hearing loss, as these may be indicative of underlying medical conditions that require prompt attention. In cases where early detection of VS is possible, it is advisable to perform resection during the second trimester of pregnancy for tumors exhibiting Koos grade IV, measuring greater than 3 cm, or displaying cystic degeneration. In late pregnancy, patients with reversible symptoms are advised to maintain their pregnancy as far as possible by conservative measures or minimally invasive procedures, provided that a balance is achieved between ensuring fetal maturation and protecting vital maternal neurological functions. In cases where the extent of nerve damage is uncertain, it is recommended that prompt delivery and surgical decompression be performed if the patient experiences a rapid loss of muscle strength on the affected side or exhibits significant deficits in the posterior group of cranial nerves.

## Data availability statement

The original contributions presented in the study are included in the article/supplementary material, further inquiries can be directed to the corresponding authors.

## Ethics statement

The studies involving humans were approved by Institutional Review Board of University of Electronic Science and Technology of China on human research. The studies were conducted in accordance with the local legislation and institutional requirements. Written informed consent for participation was not required from the participants or the participants’ legal guardians/next of kin in accordance with the national legislation and institutional requirements. Written informed consent was obtained from the individual(s) for the publication of any potentially identifiable images or data included in this article.

## Author contributions

BM: Data curation, Formal analysis, Methodology, Resources, Writing – original draft. HY: Data curation, Investigation, Methodology, Writing – original draft, Writing – review & editing. TY: Data curation, Formal analysis, Resources, Writing – review & editing. LL and ZM: Data curation, Resources, Writing – review & editing. LY: Data curation, Resources, Writing – review & editing. ZY: Data curation, Funding acquisition, Resources, Writing – review & editing. HQ: Formal analysis, Funding acquisition, Investigation, Writing – review & editing. WB: Data curation, Methodology, Writing – review & editing. WH: Conceptualization, Resources, Writing – review & editing.

## References

[1] Schoemaker MJ Swerdlow AJ Auvinen A Christensen HC Feychting M Johansen C. Medical history, cigarette smoking and risk of acoustic neuroma: an international case-control study. Int J Cancer. (2007) 120:103–10. doi: 10.1002/ijc.22272, PMID: 17019705

[2] Doyle KJ Luxford WM. Acoustic neuroma in pregnancy. Am J Otol. (1994) 15:111–3. PMID: 8109621

[3] Shah KJ Chamoun RB. Large vestibular schwannomas presenting during pregnancy: management strategies. J Neurol Surg B Skull Base. (2014) 75:214–20. doi: 10.1055/s-0034-1370784, PMID: 25072015 PMC4078191

[4] Kurowska-Mroczek E Zabek M Osuch B Stelmachów J. Therapeutic management of acoustic neurinoma during twin pregnancy: a case report. J Reprod Med. (2009) 54:393–6. PMID: 19639930

[5] Kasantikul V Brown WJ. Estrogen receptors in acoustic neurilemmomas. Surg Neurol. (1981) 15:105–9. doi: 10.1016/0090-3019(81)90023-9, PMID: 7245001

[6] Tan NC Macfarlane R Donnelly N Mannion R Tysome JR Jefferies S. A 2 and 5-year longitudinal analysis of 671 consecutive patients diagnosed with unilateral vestibular schwannoma. Otol Neurotol. (2022) 43:702–8. doi: 10.1097/MAO.0000000000003536, PMID: 35709433

[7] Gaughan RK Harner SG. Acoustic neuroma and pregnancy. Am J Otol. (1993) 14:88–91. PMID: 8424484

[8] Kachhara R Chandrika Devi CG Nair S Bhattacharya RN Radhakrishnan VV. Acoustic neurinomas during pregnancy: report of two cases and review of literature. Acta Neurochir. (2001) 143:587–91. doi: 10.1007/s007010170063, PMID: 11534675

[9] Akella S Mattson J Moreira N. Vestibular schwannoma growth during pregnancy: case report of neurofibromatosis type 2 in pregnancy. Proc Obstet Gynecol. (2019) 9:5–9.

[10] Thacker JG Wallace EM Whittle IR Colder AA. Successful excision of a giant acoustic neuroma in the third trimester of pregnancy. Scott Med J. (1995) 40:117–8. doi: 10.1177/003693309504000405, PMID: 8787111

[11] Allen J Eldridge R Koerber T. Acoustic neuroma in the last months of pregnancy. Am J Obstet Gynecol. (1974) 119:516–20. doi: 10.1016/0002-9378(74)90212-9, PMID: 4842596

[12] Beatty CW Scheithauer BW Katzmann JA Roche PC Kjeldahl KS Ebersold MJ. Acoustic schwannoma and pregnancy: a DNA flow cytometric, steroid hormone receptor, and proliferation marker study. Laryngoscope. (1995) 105:693–700. doi: 10.1288/00005537-199507000-00005, PMID: 7603272

[13] Siglock TJ Rosenblatt SS Finck F House WF Hitselberger WE. Sex hormone receptors in acoustic neuromas. Am J Otol. (1990) 11:237–9. PMID: 2399940

[14] Klinken L Thomsen J Rasmussen BB Wiet RJ Tos M. Estrogen and progesterone receptors in acoustic neuromas. Arch Otolaryngol Head Neck Surg. (1990) 116:202–4. doi: 10.1001/archotol.1990.01870020078020, PMID: 2297415

[15] Curley JW Ramsden RT Howell A Healy K Lye RH. Oestrogen and progesterone receptors in acoustic neuroma. J Laryngol Otol. (1990) 104:865–7. doi: 10.1017/S0022215100114197, PMID: 1702456

[16] Chakravarthy V Kaplan B Gospodarev V Myers H de Los Reyes K Achiriloaie A. Houdini tumor: case report and literature review of pregnancy-associated meningioma. World Neurosurg. (2018) 114:e1261–5. doi: 10.1016/j.wneu.2018.03.187, PMID: 29626688

[17] Bonfield CM Engh JA. Pregnancy and brain tumors. Neurol Clin. (2012) 30:937–46. doi: 10.1016/j.ncl.2012.04.003, PMID: 22840798

[18] Goh AY Hussain SS. Sudden hearing loss and pregnancy: a review. J Laryngol Otol. (2012) 126:337–9. doi: 10.1017/S0022215112000114, PMID: 22309465

[19] Xie S Wu X. Clinical management and progress in sudden sensorineural hearing loss during pregnancy. J Int Med Res. (2020) 48:1219670270. doi: 10.1177/0300060519870718 PMC7593668 31452412

[20] Moafi F Saberi H Bajalan Z. Large vestibular schwannoma presenting in pregnancy: a case report. Case Rep Clin Pract. (2018) 3:50–2.

[21] Beni-Adani L Pomeranz S Flores I Shoshan Y Ginosar Y Ben-Shachar I. Huge acoustic neurinomas presenting in the late stage of pregnancy. Treatment options and review of literature. Acta Obstet Gynecol Scand. (2001) 80:179–84. doi: 10.1034/j.1600-0412.2001.080002179.x, PMID: 11167216

[22] Girardelli S Albano L Mangili G Valsecchi L Rabaiotti E Cavoretto PI. Meningiomas in gynecology and reproduction: an updated overview for clinical practice. Reprod Sci. (2022) 29:2452–64. doi: 10.1007/s43032-021-00606-2, PMID: 33970444

[23] Satyarthee G. Singh S. K., "Management of Giant Vestibular Schwannoma in second trimester pregnancy–review of literature, ", Vol. 1, No. (2015), pp. 142–146.

[24] Bedard JM Richardson MG Wissler RN. General anesthesia with remifentanil for cesarean section in a parturient with an acoustic neuroma. Can J Anaesth. (1999) 46:576–80. doi: 10.1007/BF03013550, PMID: 10391607

[25] Goldbrunner R Weller M Regis J Lund-Johansen M Stavrinou P Reuss D. EANO guideline on the diagnosis and treatment of vestibular schwannoma. Neuro-Oncology. (2020) 22:31–45. doi: 10.1093/neuonc/noz153, PMID: 31504802 PMC6954440

[26] Magliulo G Ronzoni R Petti R Marcotullio D Marini M. Acoustic neuroma in the pregnant patient. Eur Arch Otorhinolaryngol. (1995) 252:123–4. doi: 10.1007/BF00168034, PMID: 7598873

[27] Tyson JE Parikh NA Langer J Green C Higgins RD National Institute of Child Health and Human Development Neonatal Research Network. Intensive care for extreme prematurity--moving beyond gestational age. N Engl J Med. (2008) 358:1672–81. doi: 10.1056/NEJMoa073059, PMID: 18420500 PMC2597069

[28] He Y Zhang M Huang C Qin X Zhang Z Wang Y. Prevalence and treatment of typical and atypical headaches in patients with Chiari I malformation: a meta-analysis and literature review. Cephalalgia. (2023) 43:2089790884. doi: 10.1177/03331024221131356 36694433

[29] He Y Zheng T Wu B Wang J. Significance of modified Clivoaxial angles in the treatment of adult Chiari malformation type I. World Neurosurg. (2019) 130:e1004–14. doi: 10.1016/j.wneu.2019.07.060, PMID: 31306845

[30] Depauw P Groen R van Loon J Peul WC Malbrain MLNG de Waele JJ. The significance of intra-abdominal pressure in neurosurgery and neurological diseases: a narrative review and a conceptual proposal. Acta Neurochir. (2019) 161:855–64. doi: 10.1007/s00701-019-03868-7, PMID: 30911831 PMC6483957

[31] Hsiao CJ Yang MJ Hung JH. Acoustic neuroma and twin pregnancy. Int J Gynaecol Obstet. (1997) 58:317–8. doi: 10.1016/S0020-7292(97)00118-5, PMID: 9286868

[32] Sharma JB Pundir P Sharma A. Acoustic neuroma in pregnancy: emergency cesarean section and definitive neurosurgery. Int J Gynaecol Obstet. (2003) 80:321–3. doi: 10.1016/S0020-7292(02)00342-9, PMID: 12628539

